# Acoustic monitoring and analyses of air gun, pile driving, vessel, and ambient sounds during the 2015 seismic surveys on the Sakhalin shelf

**DOI:** 10.1007/s10661-022-10021-y

**Published:** 2022-10-18

**Authors:** Alexander N. Rutenko, Mikhail M. Zykov, Vladimir A. Gritsenko, Mikhail Yu. Fershalov, Michael R. Jenkerson, Denis S. Manulchev, Roberto Racca, Vladimir E. Nechayuk

**Affiliations:** 1 Far East Branch, V.I. Il’ichev Pacific Oceanological Institute, Russian Academy of Sciences, Vladivostok, 690041 Russia; 2 JASCO Applied Sciences Ltd, Dartmouth, NS B2Y 4S3 Canada; 3 ExxonMobil Exploration Company, Spring, TX 77389 USA; 4 JASCO Applied Sciences Ltd, Victoria, BC V8Z 7X8 Canada; 5 Lucas, TX 75002 USA

**Keywords:** Russia, Sakhalin Island, Acoustic monitoring, Air gun sounds, Pile driving, Vessel sounds, Gray whales

## Abstract

During the summer of 2015, four 4D seismic surveys were conducted on the northeastern Sakhalin shelf near the feeding grounds of the Korean-Okhotsk (western) gray whale (*Eschrichtius robustus*) population. In addition to the seismic surveys, onshore pile driving activities and vessel operations occurred. Forty autonomous underwater acoustic recorders provided data in the 2 Hz to15 kHz frequency band. Recordings were analyzed to evaluate the characteristics of impulses propagating from the seismic sources. Acoustic metrics analyzed comprised peak sound pressure level (PK), mean square sound pressure level (SPL), sound exposure level (SEL), *T*_100%_, *T*_90%_ (the time intervals that contain the full and 90% of the energy of the impulse), and kurtosis. The impulses analyzed differed significantly due to the variability and complexity of propagation in the shallow water of the northeast Sakhalin shelf. At larger ranges, a seismic precursor propagated in the seabed ahead of the acoustic impulse, and the impulses often interfered with each other, complicating analyses. Additional processing of recordings allowed evaluation and documentation of relevant metrics for pile driving, vessel sounds, and ambient background levels. The computed metrics were used to calibrate acoustic models, generating time resolved estimates of the acoustic levels from seismic surveys, pile driving, and vessel operations on a gray whale distribution grid and along observed gray whale tracks. This paper describes the development of the metrics and the calibrated acoustic models, both of which will be used in work quantifying gray whale behavioral and distribution responses to underwater sounds and to determine whether these observed responses have the potential to impact important parameters at the population level (e.g., reproductive success).

## Introduction

**Fig. 1:**
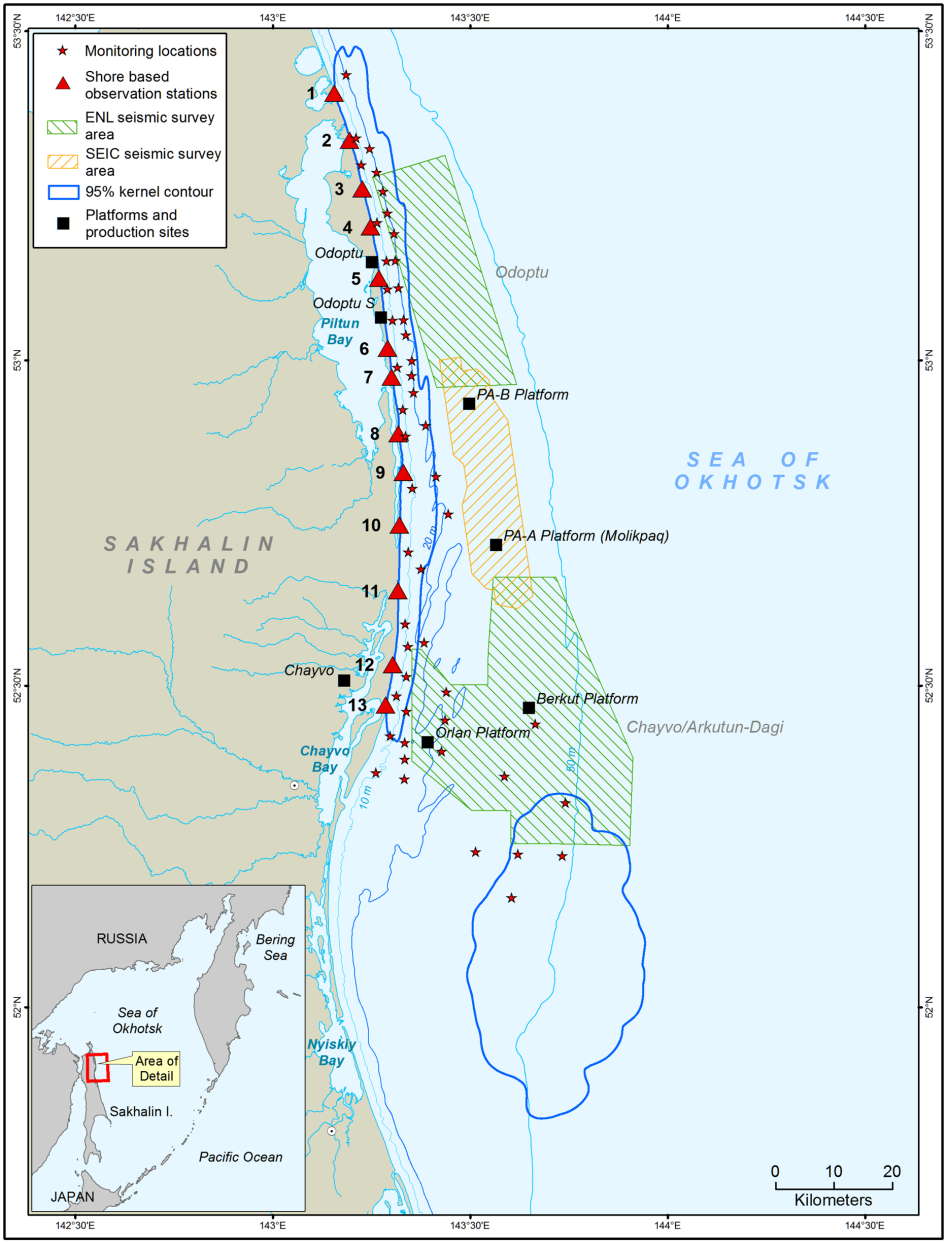
Map of the study area showing the 2015 seismic survey areas, the locations at which acoustic monitoring was conducted, and the 95% kernel contours for the nearshore and offshore gray whale feeding areas

The shallow water region of the northeast Sakhalin shelf, starting south of the mouth of Piltun Bay and extending northwards up the Sakhalin coast, is the most important known summer and fall feeding area for the Korean-Okhotsk (western) gray whale (*Eschrichtius robustus*) population. The historically known nearshore gray whale feeding grounds are in water depths of < 20 m, and gray whales occur on average approximately 1.5 km from shore (Gailey, 2013; Gailey et al., 2016; Muir et al., 2015, 2016) (Fig. 1). In 2001, a second gray whale feeding area (the offshore feeding area) was discovered in deeper water (30–60 m), approximately 20 km southeast of the mouth of Chayvo Bay (Johnson et al., 2007; Maminov & Yakovlev, 2002).

The Korean-Okhotsk (western) gray whale population is currently listed as “Category 1 status” in the Red Book of Russia and, at the time of the survey, as “Critically Endangered” by the International Union for the Conservation of Nature (IUCN) (Cooke et al., 2018). Based on a recent re-evaluation, including gray whales observed not only off Sakhalin but also off Kamchatka, the status of the western gray whale on the IUCN Red List was changed to “Endangered” in 2018 (Cooke et al., 2018).

The Sakhalin-1 license area fields Odoptu, Chayvo, and Arkutun-Dagi are located near gray whale feeding areas. In the summer of 2015, Exxon Neftegas Limited (ENL) acquired 4D seismic surveys in these three areas using three seismic vessels*.* Sakhalin Energy Investment Company (SEIC) conducted a 4D seismic survey in their Piltun-Astokh license area situated between the Chayvo and Odoptu fields during the same period. Other sources of anthropogenic sound that could have reached the feeding areas included the emplacement of onshore foundation piles (metal pipes driven into the ground to support facilities) at the Odoptu south well site and Chayvo production site as well as vessels operating in the region (Fig. 1).

Concerns about the potential effects of anthropogenic underwater sounds prompted an extensive monitoring, mitigation, and research program (Aerts et al., 2022). This paper describes efforts undertaken as part of that program to characterize the soundscape before, during, and after operations that potentially ensonified the gray whale feeding areas. One of the environmental questions that the program endeavored to address was whether the criterion adopted in past seismic surveys for mitigating behavioral effects is in fact optimal for minimizing disturbance to the feeding activity of western gray whales. Another key subject of investigation was whether sound generated by these operations could cause a population level effect on the western gray whale.

From June to October 2015, 40 Autonomous Underwater Acoustic Recorders (AUARs) were deployed 126 times and acquired 4312 days of acoustic measurements. The recordings were processed post-season to detect, identify, and characterize impulses generated by seismic surveys or pile driving activities with a received peak sound pressure level above 105 dB re 1 µPa (a threshold below which impulse characterization would be problematic). Recordings were also used to characterize vessel traffic and ambient sounds. Processed data were then used to calibrate acoustic models that could be applied to characterize acoustic exposure of whales in the study area. Model outputs included (1) exposure levels at 300-s intervals on a 34 × 130 km gray whale distribution grid with a 1 km^2^ cell size and (2) time series of estimated exposures with a 30-s step size along individual gray whale tracks in the nearshore feeding area provided by shore-based marine mammal observers.

In work described by Gailey et al. (2022a, b) and Schwarz et al. (2022), these model outputs are incorporated as potential explanatory variables in multivariate analyses examining potential influences of anthropogenic sounds on observed gray whale behavior, distribution, and energetics in the study area during 2015.

## Methods

### Equipment

In 2015, thirty-nine newly designed AUARs produced by the Pacific Oceanological Institute, Far East Branch, Russian Academy of Sciences (POI), and one earlier design AUAR were deployed over the study period at 48 distinct acoustic monitoring sites (Fig. 1). The AUARs use a 24-bit delta-sigma analog-to-digital converter (ADC) to acquire autonomous acoustic pressure measurements in the frequency range from 2 Hz to 15 kHz with a dynamic range of 145 dB. A low-power controller allows the systems to operate continuously for more than 6 months. The strong tidal currents of up to 1.5 m/s and the shallow water depths (≤ 20 m) at most of the acoustic monitoring stations necessitated the development of recording equipment especially suited to that environment. Filtering within the hydrophone preamplifier gives a response in the frequency band from 10 to 15,000 Hz of approximately 63 dB re 1 μV/Pa, while the response at 2 Hz is more than 40 dB lower. This response, at the natural sensitivity of the hydrophone ceramics of 1 mV/Pa, provides the dynamic range required for making acoustic measurements in the presence of flow and swell noise (Borisov et al., 2008). The preamplifier of the GI-50 hydrophone and the 24-bit delta-sigma ADC used in the AUARs yield an instantaneous dynamic range of more than 120 dB for a 1-Hz tonal signal (Rutenko et al., 2015).

### Estimating impulse characteristics 

**Fig. 2:**
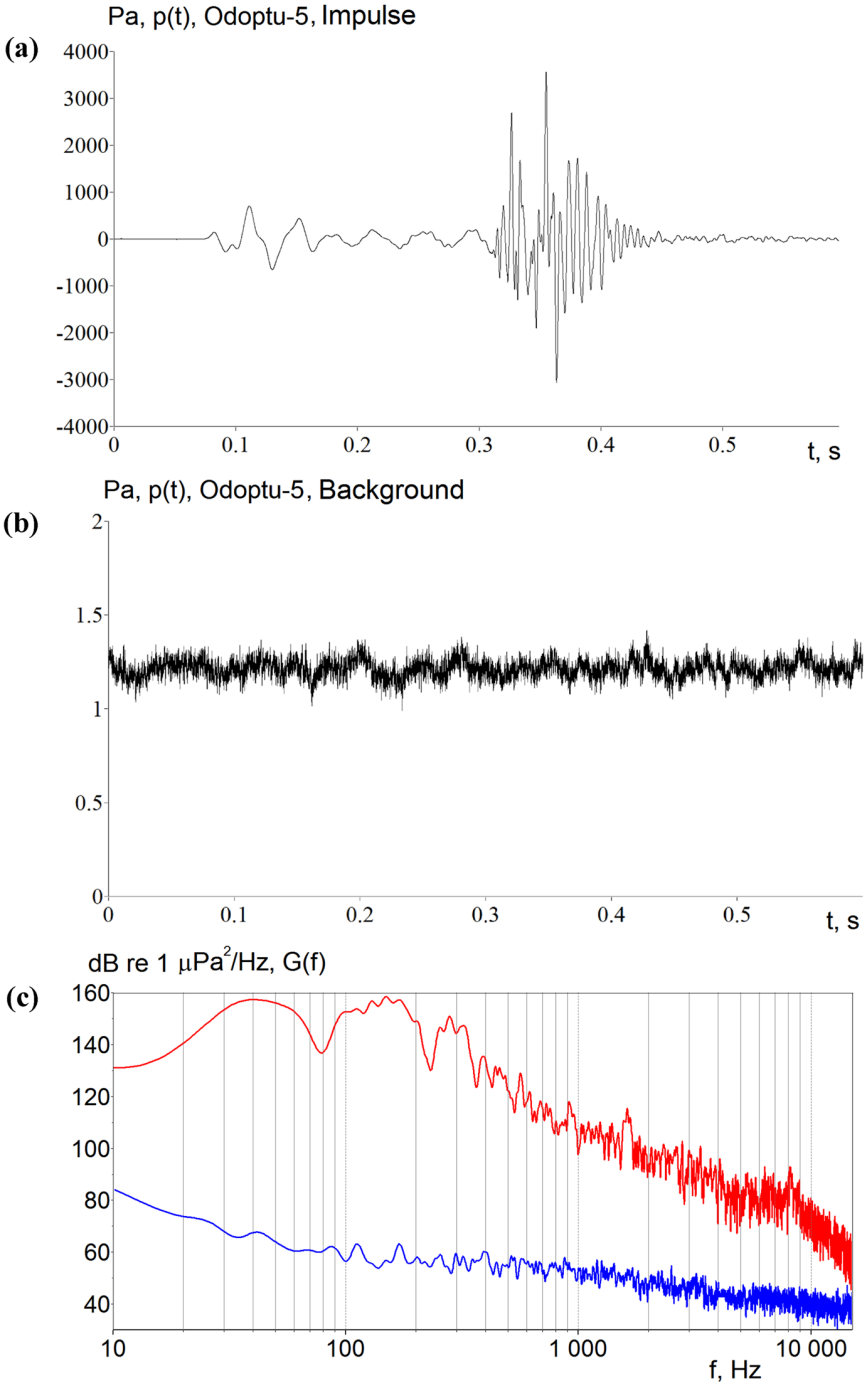
Acoustic measurements of **a** a seismic impulse acquired by an Autonomous Underwater Acoustic Recorder (AUAR) deployed at a depth of 20 m **b** the background signal under calm conditions and **c** spectral plots of the seismic impulse (red) and background (blue) measurements

Figure 2 illustrates the signal response characteristics of the AUARs using acoustic data acquired near the bottom in a water depth of 20 m near the Odoptu field. Specifically, Fig. 2 shows the time-domain plots of a seismic impulse emitted by a 2400 in^3^ air gun array source at a distance of 1.3 km (a) and of a background signal recorded under calm conditions in the absence of meaningful sounds from seismic surveys, pile driving, and vessels near the AUAR (b). The spectral (frequency domain) distributions of the two measured signals are also shown (c). In this example, the peak pressure of the air gun array impulse reached about 3.5 kPa. Pressure variations due to the energy carried by the seismic (ocean bottom propagated) precursor and acoustic (water propagated) modes are clearly discernible in the time domain, beginning at about 0.1 and 0.3 s, respectively (Fig. 2a). Three power peaks can be identified in the spectral plot at frequencies of 20–70 Hz, 90–200 Hz, and 250–350 Hz (Fig. 2c).

To estimate the received sound levels on a whale distribution grid and along whale tracks, all impulses from seismic surveys and onshore pile driving received at the recording stations, including weaker ones, had to be analyzed. This required a sophisticated characterization of the received impulse waveforms.

**Fig. 3:**
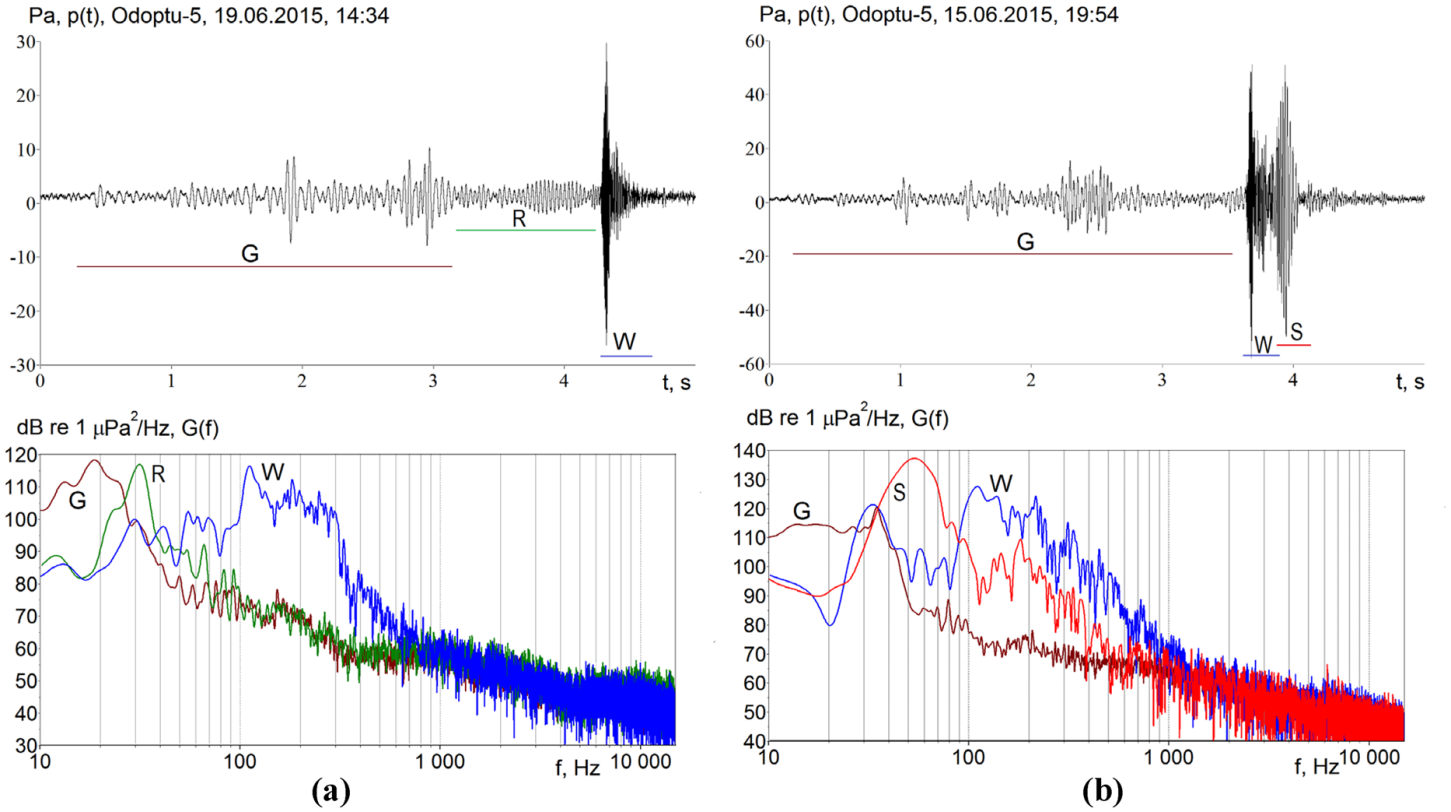
Time and frequency domain plots of data recorded in a water depth of 20 m at the Odoptu–5 acoustic monitoring station and corresponding to seismic impulses emitted at a depth of 6 m at a distance of **a** 18 km and **b** 14 km from the station. In both examples, colors and letter labels represent wave arrival time periods: G, seismic (ground-borne) waves; R, resonance channel waves; W, water-borne waves; S, surficial sediment waves

As the length of the propagation path increases beyond 10 km and the orientation of the source-receiver transect becomes more parallel to the shore, the acoustic impulse at the monitoring point takes on a complex temporal and spectral structure that was influenced by the specific characteristics of seismic propagation in the seabed (Fig. 3). These spatial heterogeneities strongly influence the propagation of low-frequency (below 40 Hz) energy captured by a spatial resonance channel for waves with a frequency of 31 Hz, as illustrated by the acoustic pressure variations in time interval R (Fig. 3a top) and their spectra (Fig. 3a bottom). Seismic waves with frequencies from 10 to 26 Hz (G) penetrated deeper into the seabed, propagated at velocities around 1900 m/s, refracted, and coupled back into the water layer before the arrival of the water modes (W). From a biological perspective, the water modes carry the most acoustic energy at the frequencies believed to be relevant to baleen whale hearing.

Energy trapped in the resonance channel formed in a surficial sediment layer with a sound propagation velocity lower than in water can cause substantial amplitude variations and interfere with the direct (acoustic) impulse, as illustrated in the example provided in Fig. 3b (top). The time domain and spectral plots from Fig. 3b show that in time interval S, the relatively narrow-band impulse has a single power peak at frequencies of between 40 and 60 Hz (red plot).

Since the acoustic energy at the receiving point is spread out in time due to complex propagation through bottom and water modes, the characteristics of received seismic impulses are analyzed over a 6-s time window to capture both the seismic (G) (precursor) and acoustic (W) (waterborne) modes; 6 s is less than the repetition rate for the seismic impulses, which ensures that, in general, only a single impulse from a given source is included in the analysis. However, in some cases, multiple-source scenarios might need additional screening to exclude extraneous arrivals.

While evaluating the characteristics of high-amplitude impulses is straightforward, the objective of the analysis was to determine the characteristics of all impulses whose peak sound pressure level exceeds ambient by 6 dB or greater. The analysis also required that the source identity and location of each impulse be determined; this task was complicated by the simultaneous presence of more than one possible source for the impulses recorded at a specific station. Up to three seismic sources could at times operate simultaneously in production mode in 2015; additionally, ENL seismic vessels used a mitigation air gun during line changes. For this study, we developed an analysis procedure capable of successfully determining the source, location, and characteristics of all the impulses with a signal-to-noise ratio ≥ 6 dB (Rutenko et al., 2019).

The acoustic data collected in 2015 contained approximately 15 million impulses that required evaluation. Software (developed by POI) automatically searched for qualifying impulses recorded at each of the AUARs and estimated their characteristics. The core metrics evaluated for each impulse were peak sound pressure level (PK), mean square sound pressure level (SPL), sound exposure level (SEL), *T*_100%_, *T*_90%_ (the time intervals that contain the full and 90% of the energy of the impulse), and kurtosis, because these metrics are considered relevant when evaluating marine mammal responses.

Once the characteristics of the impulses had been estimated, a separate set of quality control programs was used to check the results, including the correct identification of the seismic vessel that generated each impulse.

## Results

### Modeling the sound field from seismic surveys

The modeling performed for the post-season data analyses was substantially more complex and sophisticated than the estimations of acoustic levels conducted prior to the season in support of the implementation of the mitigation plan (Rutenko et al., 2022). Having a considerable body of ancillary data available from the monitoring of operations, in addition to archival recordings of the received acoustic levels at 48 locations, allowed a far more comprehensive model verification and compensation than had been possible for any previous studies in this region (see Racca et al., 2015). The different analyses required distinctions between instantaneous estimates of sound levels along individual gray whale movement tracks for behavioral response studies and sound energy estimates averaged within 1 km^2^ grid cells for gray whale distribution analyses (Gailey et al., 2022a, b). The former, henceforth denoted as whale track exposure (WTE) modeling, required high-resolution modeling both temporally and spatially because the potential response of a whale to received acoustic levels could be influenced by transient and localized features of the sound field. The latter, denoted as grid exposure (GE) modeling, required a lower resolution estimation in space and time to provide meaningful trends potentially associated with longer term responses of gray whales to the soundscape in different regions.

The parameterization of the seafloor properties used in the propagation model was adjusted through testing of several geo-acoustic profiles that were plausible variations on the historically tuned values (Hannay & Racca, 2005). The parametrization that yielded the smallest statistical mismatch between measured and estimated impulse levels considering the entire set of monitoring data was selected as the standard profile and used for the entire modeling region. For the sound propagation properties in the water column (vertical Sound Speed Profile, SSP), the approach was different for the WTE and GE cases. For WTE, the individual SSP measurement(s) from a variety of provenances (the seismic survey vessels, acousticians aboard the R/V *Igor Maksimov*, and other opportunistic sources) that most closely matched the timing and area of the whale observation were used to parametrize the model. For GE, four averaged SSPs — one each for two areas (Odoptu and Chayvo) and two time periods (June-July and August–September) — were compiled from all corresponding SSP measurements and used in the modeling over the grid region and temporal span of the operations.

Navigational information for the source vessels was used for the accurate positioning of the air gun source arrays to model each of the seismic surveys, which included SEIC’s Piltun-Astokh 4D survey for which the acquisition schedule overlapped with both the Chayvo and Arkutun-Dagi surveys. Additionally, a detailed schedule of geophysical source activity for each operation was generated in support of the acoustic modeling of the seismic surveys.

The records of acoustic impulses received at the AUARs were the foundation not only of source activity and timing data but also, and primarily, of impulse levels and other metrics at multiple locations that would be used in the calibration and adjustment of model estimates over the relevant study region. The acoustic characteristics for detected impulses at each receiver were preconditioned by grouping the impulses from each seismic source separately in 60-s bins on a common time basis. In each 60-s bin, the following statistics were computed: number of impulses, median SEL, median SPL, median and maximum PK, median SEL-SPL difference, median SEL-PK difference, and mean kurtosis. These 60 s values were used for model estimate compensation and conversion in the exposure modeling (Gailey et al., 2022a).

The modeling algorithm for this study was tuned in its handling of reference data gaps not to favor overestimating the acoustic exposure of the whales (against the common approach used when assessing risk), as this would lead to underrating their response sensitivity. Modeling results to be used in behavioral analysis therefore lean on the side of false negatives (the model does not predict a sound event that did take place) and most-likely sound exposure estimation, rather than false positives (the model predicts a sound event that did not take place) and maximized sound exposure estimation. The latter bias would lead to the conclusion of lesser sensitivity to sound than would be the actual case because responses (or absence thereof) would be associated with overrepresented exposure. Thus, if no objective information on the operating mode or the impulse repetition rate was available at a given time (other than the source was active), the operating mode for the mitigation gun (used only in ENL operations) assumed a default repetition rate of 1.5 impulses per 60 s; if the source was known to be in production mode, a repetition rate of 7.5 impulses per 60 s was assumed.

The airgun array source model (AASM; MacGillivray, 2006) was used to predict the pressure signatures and directional source levels of the full air gun arrays (2400 in^3^ for the ENL seismic surveys and 2888 in^3^ for the SEIC survey) and the mitigation air gun (70 in^3^ volume) (used only on ENL operations). The SEL of impulses radiated by the sources was modeled from the computed spectral source levels with the parabolic equation (PE) based marine operations noise model (MONM; Austin & Chapman, 2011). MONM is based on the widely accepted RAM code (Collins, 1993), modified to account for shear wave losses at the seafloor by applying a complex multiplicative factor to the seabed density (Zhang & Tindle, 1995). This approach is more than five times faster than code that treats shear wave propagation in a robust sense, yet it produces results that are nearly identical to the reference approach for uniform, low shear-speed, shallow-water environments with silt and sand bottoms (Hannay & Racca, 2005). The PE code does not model potential interface waves near the seafloor at frequencies of a few hertz. Sound propagation loss (PL) was computed in 1/3-octave bands, and received sound level was computed by applying the PL to the source level in each of the bands and summing to obtain the broadband value. The modeling was open ended (uncompensated) by default but was adjusted to match more closely the measured SEL for the time bin of the modeled impulse provided that monitoring data from acoustic stations were available near the model target location. The SPL output of production modeling was based on the estimated SEL converted using the SEL-SPL offset for the time bin of the impulse if available at nearby AUARs; if unavailable, a default conversion factor based on an empirical SEL-SPL vs. range function (GE case) or constant + 3 dB offset (WTE case) was applied. The PK output was similarly based on the estimated SEL and the tabulated SEL-PK offset if available, or a default empirical range-dependent offset for the GE modeling and constant offset of + 15 dB for the WTE modeling. The empirical range-dependent offset functions were defined through statistical analysis of the impulse characteristics data from the acoustic stations. For the kurtosis, no modeling was used; rather, the estimate was a geometrically weighted average of the kurtosis values for the relevant time bin at nearby acoustic stations. This type of output was used in the post-season analysis to facilitate the attribution of received impulses to a given seismic vessel and to optimize the model correction approach for available data.

For WTE estimation, the modeling time window was defined by the duration of each whale track (from a few minutes to several hours); the position of the whale along the track line was sampled every 30 s (see Gailey et al., 2022a, b for track line and resampling procedures). The PL was computed running MONM along direct radials from the current source position to the whale location (taking the maximum-over-depth value of the estimated SEL results) and to the four nearest AUARs within 15 km of the whale position to assess the model accuracy through comparison with the measurements and to yield a SEL adjustment. For efficiency, if the whale-source range at the next time step did not change by more than 1% from the previously modeled positions, the same PL value was used; the impulse information (impulse count, measured SEL), however, was always taken for the actual time window. The maximum range considered for modeling impulse levels from seismic surveys was 70 km. The source level selection was based on the operational condition of the seismic vessel at the current time step (whether in full array production mode or mitigation mode).

**Fig. 4:**
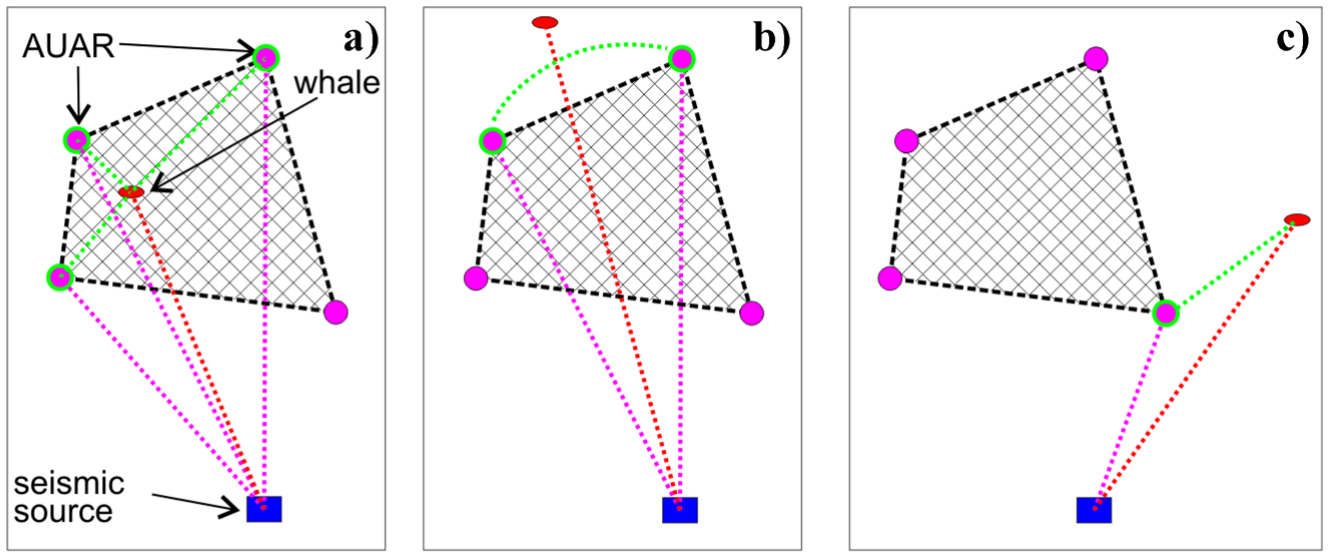
Geometry of calculating a correction for three cases of whale-AUAR co-location: **a** Inside the group of AUARs **b** outside the group, but within sector of two AUARs and **c** outside the sector

The correction to the modeled SEL value for the whale position was calculated by a method that depended on the relative position of the AUAR and the whale as shown in Fig. 4. If the whale position was within the group of acoustic stations (Fig. 4a), the correction was calculated using triangulation based on three points. If it was outside the group but within an angular sector formed by two stations (Fig. 4b), the correction was computed by linear interpolation based on azimuth. If it was totally outside the cluster of stations (Fig. 4c), the correction was estimated from the nearest AUAR in azimuth. The same algorithm was used to estimate the SEL-SPL and SEL-PK conversion offsets at the whale position using calculated offsets at the AUARs and for the estimated kurtosis of the impulse. If no suitably located AUAR existed or there were no signal data for a given source at the selected AUAR, the modeled SEL was not corrected, the default offsets were used to calculate SPL and PK, and the kurtosis of the impulse remained undefined. The cumulative SEL over the 30-s interval was based on the per-impulse SEL and the number of impulses, taken from the seismic production logs if available. In the absence of positioning records for the period of interest, the impulse count information at the three AUARs closest to the source was considered. If no information was available that explicitly defined the number of impulses for the 30-s interval, but the operational schedule indicated that the seismic source was active, the default impulse count was used (7.5 impulses per 60 s for operations and 1.5 impulses per 60 s for mitigation air guns).

**Fig. 5:**
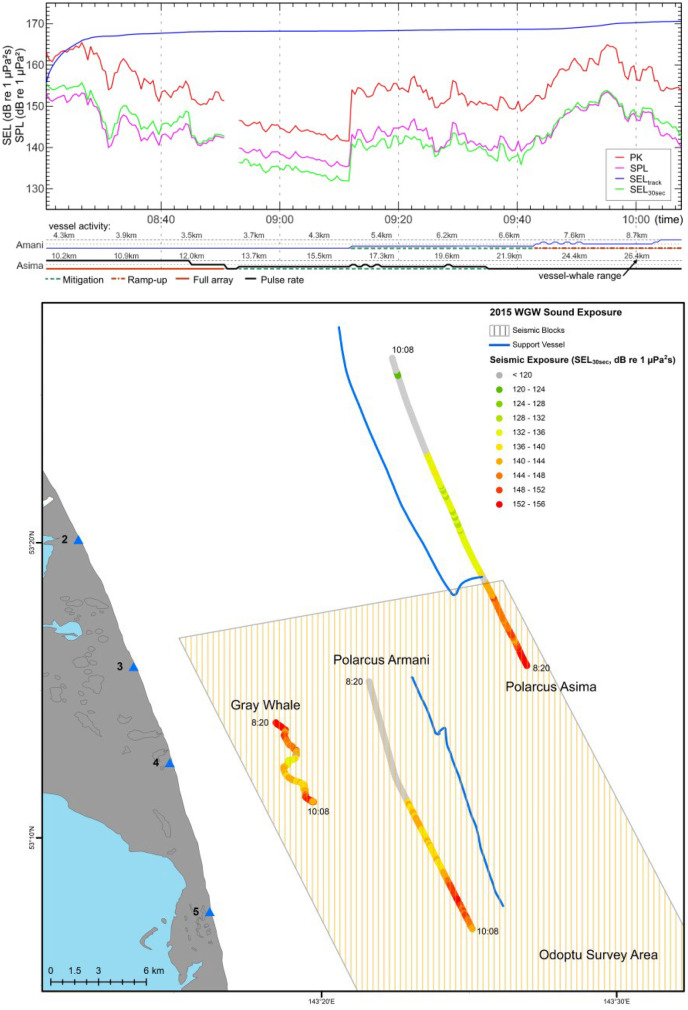
Acoustic exposure based on modeling results for a whale track. Top panel: PK, SPL, SEL from the start of the track, and SEL for each 30-s window. Shown below the plots are the names of active seismic vessels, their mode of operation (green and red lines), pulse rate (blue line), and range to the whale. Bottom panel: map rendering of SEL in 30-s windows as received at the whale track and its contribution from each of the seismic vessels (overlaid on the track of each vessel but representing the level at the whale)

The modeling results were output with the following information provided for each point on the whale’s track (for each source separately): source coordinates, range between source and whale, seismic source operating mode, number of impulses, accumulated SEL over 30 s, SPL, PK, kurtosis, SEL correction, and the correction calculation method used (based on 3, 2, or 1 reference acoustic stations or uncorrected modeling). As an example, Fig. 5 provides representations of the modeled exposure for various metrics on a whale track, both as time-series plots and as a color-coded rendering of the received exposure level at the whale track and its additive contributions from each of the seismic vessels (see Gailey et al., 2022a).

The GE estimation from the seismic survey activities spanned from 10 June to 24 September 2015 and was conducted for two target regions — the nearshore feeding area (NFA) and the offshore feeding area (OFA) — using different resolutions and correction approaches depending on the analytical requirements that the results would support and the availability of reference field acoustic data.

For the NFA GE, the estimation was performed for a grid of 1 × 1 km cells, with exposure values defined for every 300-s period. The computation involved two phases. First, a framework of source sites with 500 m spacing was created at which PL modeling was performed along a fan of radials. The framework of the pre-modeled sites spanned the area in which acoustic sources (both seismic and vessels) were active. The fan of modeling radials was formed to cover the entire target grid for which exposure was to be estimated. The angle between radials was adaptively set between a minimum of 1.4° and a maximum of 3° depending on the reach between the source position and the farthest boundary of the target grid, with the aim to keep the separation between the ends of adjacent radials within 1000 m. The PL modeling was performed in 1/3-octave bands using MONM over the 16–500 Hz frequency range. As previously mentioned, the PL pre-modeling at each source location was performed with two water SSPs, one for June-July and one for August–September, and these seasonal SSPs were different for the northern and southern half of the overall modeling region. Having defined the framework of PL pre-modeled sites, in the second phase the received levels were estimated on a 250 × 250 m cell size computational grid at 60-s intervals, synchronous with the binned impulse measurements from the AUARs. At each time step, the PL data were taken from the pre-modeled site nearest to the position of active seismic, and the RL values on the computational grid were calculated for each source considering its operational mode, air gun array orientation, and number of impulses over the step period, applying where possible a SEL adjustment and SPL and PK conversion based on the impulse measurements as described further below. The algorithm for selecting the operational mode of the seismic source and the impulse count resembled the one utilized in WTE modeling, with the addition of the “ramp up” mode. Ramp up is the period over which the number of air guns is progressively increased to gradually raise the level of sound exposure; these times were manually detected using the impulse analysis and the marine mammal observer schedule; to model ramp up, the broadband output of the production array source was initially reduced by 12 dB and linearly raised to full at the end of the period.

The SEL correction and SPL/PK conversion were performed at each 60-s step as follows. For a given seismic source, the SEL at each AUAR (reference point) was estimated using the same framework of PL pre-modeled sites, and the mismatch with the measured SEL for that time step was calculated for that reference point. The mismatch was spatially interpolated along radials extending from the source through the AUAR locations assuming it to be zero at the source position and clamped at the calculated value at the reference point and beyond it. Between these primary radials of mismatch values extending to actual field receiver locations, a polar grid of additional radials was created by interpolation between those with the reference point correction to densify the coverage; the radial array of values was then resampled over the matrix of computational grid points to produce a correctional grid for the modeled RL values. The same approach was followed to produce conversion grids for SEL-SPL and SEL-PK using the difference between those metrics measured at the reference points to adjust the default values along the radial transect.

**Fig. 6:**
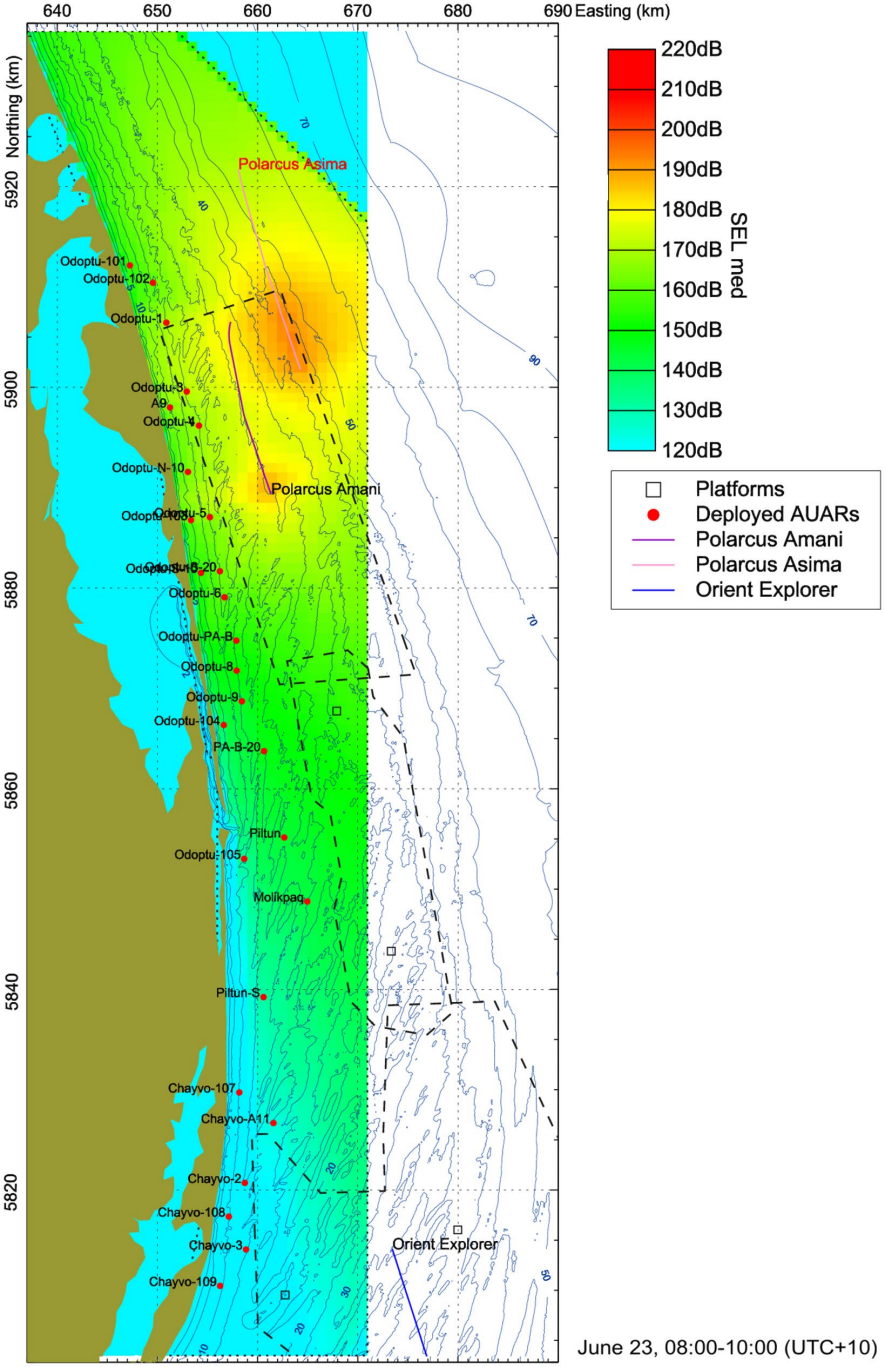
Modeled median SEL exposure for a 2-h period. The outlines of the survey areas, seismic vessel tracks, and locations of the AUARs (for the period) are shown

The SEL cumulative value over the 300-s output period was built up on the computational grid by summing the exposure from each 60-s step, while the SPL and PK values were set at the maximum values from the 60-s intervals. The output grid (1 × 1 km cell size) was generated by taking the median and maximum of the SEL, SPL, and PK values over the 4 × 4 underlying cells (250 × 250 m size) of the computational grid and assigning the resulting values to the centroid of the larger cell. Figure 6 provides an example of the GE modeling output for a 2-h period, calculated as the aggregate of twenty-four median SEL 300-s period grids.

For the OFA, which covered a region of 40 × 80 km in which no systematic field sampling of received sound levels was available, the estimation was performed for a coarser output grid of 10 × 10 km cells (32 in all); this was deemed adequate for the resolution of the whale distribution data available over this area. As was the case for the NFA estimation, the acoustic values were first estimated on a finer computational grid with 2 × 2 km cell size. The calculation of the pre-modeled PL framework on the computational grid was performed identically to the NFA case. Because of the lesser requirement for precision associated with this analysis, however, some computation approximations were made in applying the PL framework to estimating received levels compared to the approach used for the NFA; in particular, the source location was assumed to be at the center of the closest computational grid cell instead of translating the framework to match the precise position of the source vessel. The precise orientation of the source array obtained from navigational data was, however, used also in this context to obtain precise directional source levels. Most importantly, the absence of a network of AUARs to provide reference measurements over the OFA region meant that all sound exposure modeling was open ended; that is, the modeling for the OFA was not subjected to compensation based on field-acquired data. For the conversion from SEL to SPL and PK, the same default offsets used in the NFA estimations in case of missing reference data were used.

The SEL cumulative value over the 1-h output period was built up on the computational grid by summing the exposure from each 60-s step, while the SPL and PK values were set at the maximum values from the 60-s intervals. The output grid (10 × 10 km cell size) was generated by taking the median and maximum of the SEL, SPL, and PK values over the 5 × 5 underlying cells (2 × 2 km size) of the computational grid and assigning the resulting values to the centroid of the output grid cell.

### Modeling the sound field from onshore foundation pile driving

Onshore foundation piles were intermittently driven at the Odoptu and Chayvo camps located on the northeast Sakhalin shelf in 2015; pile driving sometimes occurred concurrently with seismic exploration. Each blow from a pile driver generates an acoustic field that propagates into the near-shore part of the Sakhalin shelf and substantially changes the local sound levels (Rutenko et al., 2016).

**Fig. 7:**
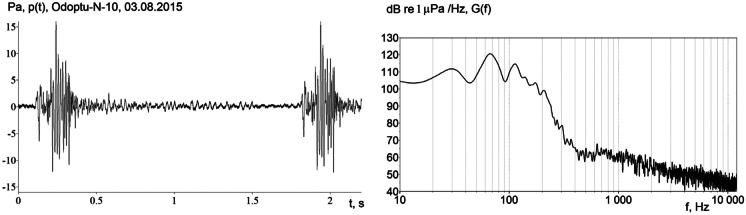
Time and frequency domain plots of impulses recorded near the seafloor at acoustic monitoring location Odoptu-N-10 during onshore foundation pile driving

A blow from a pile driver on the end of an emplaced pile excites seismic impulses that couple into the water layer as low-frequency (below 300 Hz) acoustic impulses with a period of approximately 1.8 s; the amplitude of these impulses can reach 15 Pa 1.5 km directly offshore (Fig. 7). The modeling was based on the solution of the mode parabolic equation (MPE) and incorporates the elastic properties of the seafloor and the hydrology of the water layer (Trofimov et al., 2015), calibrated using field measurements. The acoustic field was estimated in the vertical plane using an adiabatic approximation for propagating normal modes and in the horizontal plane in angular segments based on the narrow-angle parabolic equation.

Foundation piles are cylindrical steel pipes with diameters of 530 or 762 mm and lengths of 17 or 20 m. Installation of foundation piles is a multi-stage process. Initially a borehole with a diameter slightly greater than the diameter of the pile to be installed is drilled to a depth ranging from 3 to 5 m. The pile is then placed in the pre-drilled hole. A pile driver is used to install the pile to the design depth or design resistance, which in this case is within the range of 14 to 19 m. The time required to drive one pile is 7 to 23 min. Foundation piles were driven at both the onshore Odoptu S well site and the Chayvo production site (S), and AUARs recorded synchronous acoustic pressure measurements near the seafloor.

Impulses recorded at AUAR locations during the installation of foundation piles contained predominantly low-frequency energy between 10 and 50 Hz. Energy transmitted by the pile into the ground propagates as a low-frequency seismic impulse into the offshore shelf, where it couples into the water layer. The propagation losses resulting from the seismic transmission caused a sharp decrease in the power spectral density level of the impulses to background at frequencies above 50 Hz at all AUAR locations except those directly offshore.

A 3D geoacoustic waveguide consisting of a sub-bottom elastic layer with continuous distributions of elastic parameters and a water layer was constructed for the model. The model used bathymetry data from actual soundings and the measured sound velocity distribution in the water layer. The sub-bottom layer was estimated to be a sediment layer with a linear distribution of acoustic parameters and a change in the gradient of the elastic parameters part way through the layer; these acoustic parameters were calibrated using acoustic data recorded at each monitoring location. Analysis of the data recorded at a reference location directly offshore from the pile showed that the SEL of these impulses varies substantially with the emplacement depth of the pile as it is driven. We therefore decided to model the impulses generated by a single pile using three source functions that depend on the emplacement depth of the pile. Each of the source functions was estimated for point sources (Manulchev, 2016) S1, S2, and S3 at depths of 6, 9, and 12 m, respectively.

Onshore foundation piles were driven within a rectangular site extending 200 m west to east and 300 m north to south. The center of site S was 230 m from the coast. Preliminary estimates showed that moving the location of the source within this rectangular working area did not substantially alter the modeled SEL estimate of the acoustic field in the near-shore area. This was tested by estimating the SEL from the corners and center of the area in the frequency range from 10 to 140 Hz, giving a difference of less than 0.24 dB. This stability made it possible to model all piles using a single location (S) at the center of the rectangular area, regardless of the actual location of the pile being driven within the area.

**Fig. 8:**
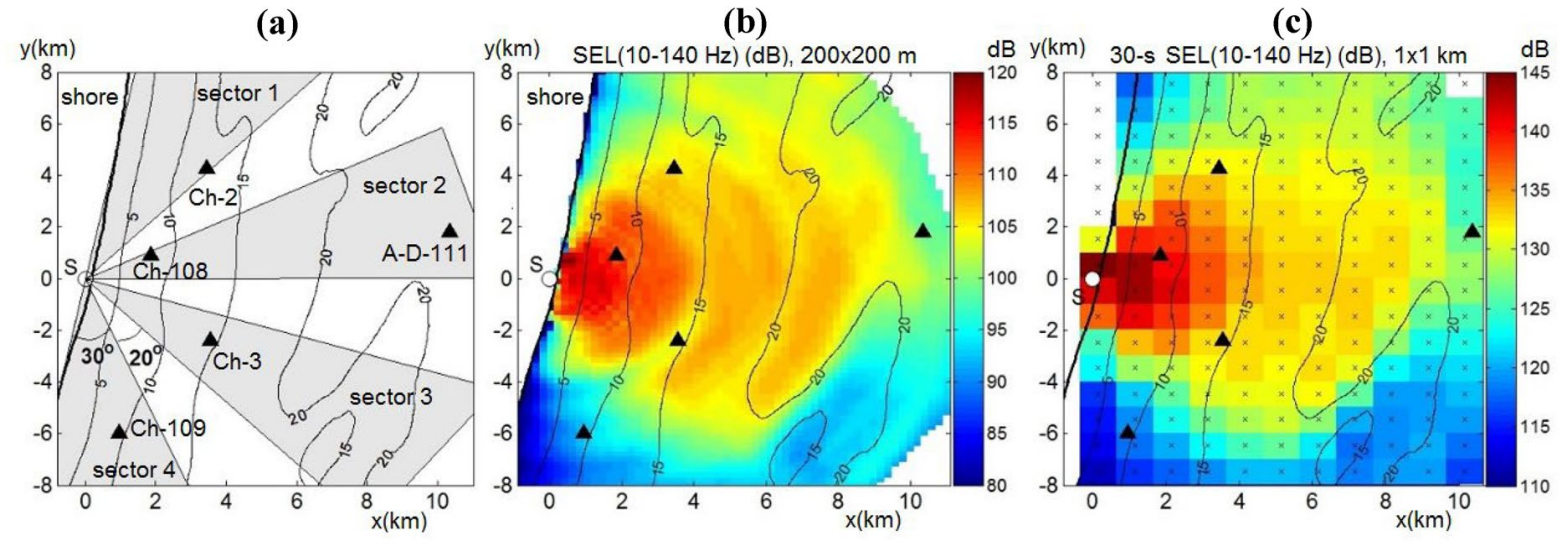
**a** Arrangement of the four narrow-angle segments used in pile driving models **b** Spatial distribution of modeled median over depth SEL(10–140 Hz) values from driving pile CN-151, averaged over a 200 × 200 m grid and **c** median-over-depth cumulative SEL(10–140 Hz) values on a 1 × 1 km grid over the installation of the pile

When solving the mode parabolic equation, estimated values for the acoustic field in the horizontal plane are only accurate in a narrow-angle segment. Since the sound source was onshore and propagates offshore, the propagation of an impulse from pile driving into a 180° area should be calculated. Therefore, we divided this area into four equidistant narrow-angle sectors, in each of which the SEL(*x,y,z*) values were calculated (Fig. 8a); the boundaries of the outside segments align with the shoreline. To determine the energy of the acoustic field at all points in the waveguide, an interpolation operation was performed at locations for which there were no calculated SEL(*x,y,z*) values..

The narrow angle segments were bounded by an angle of 30°, and the angle between the segment boundaries was 20° (Fig. 8a). Comparisons of the modeled and recorded acoustic fields show that the modeling accurately reflects the three-dimensional heterogeneities in the waveguide and bathymetric effects (Fig. 8b, c).

**Fig. 9:**
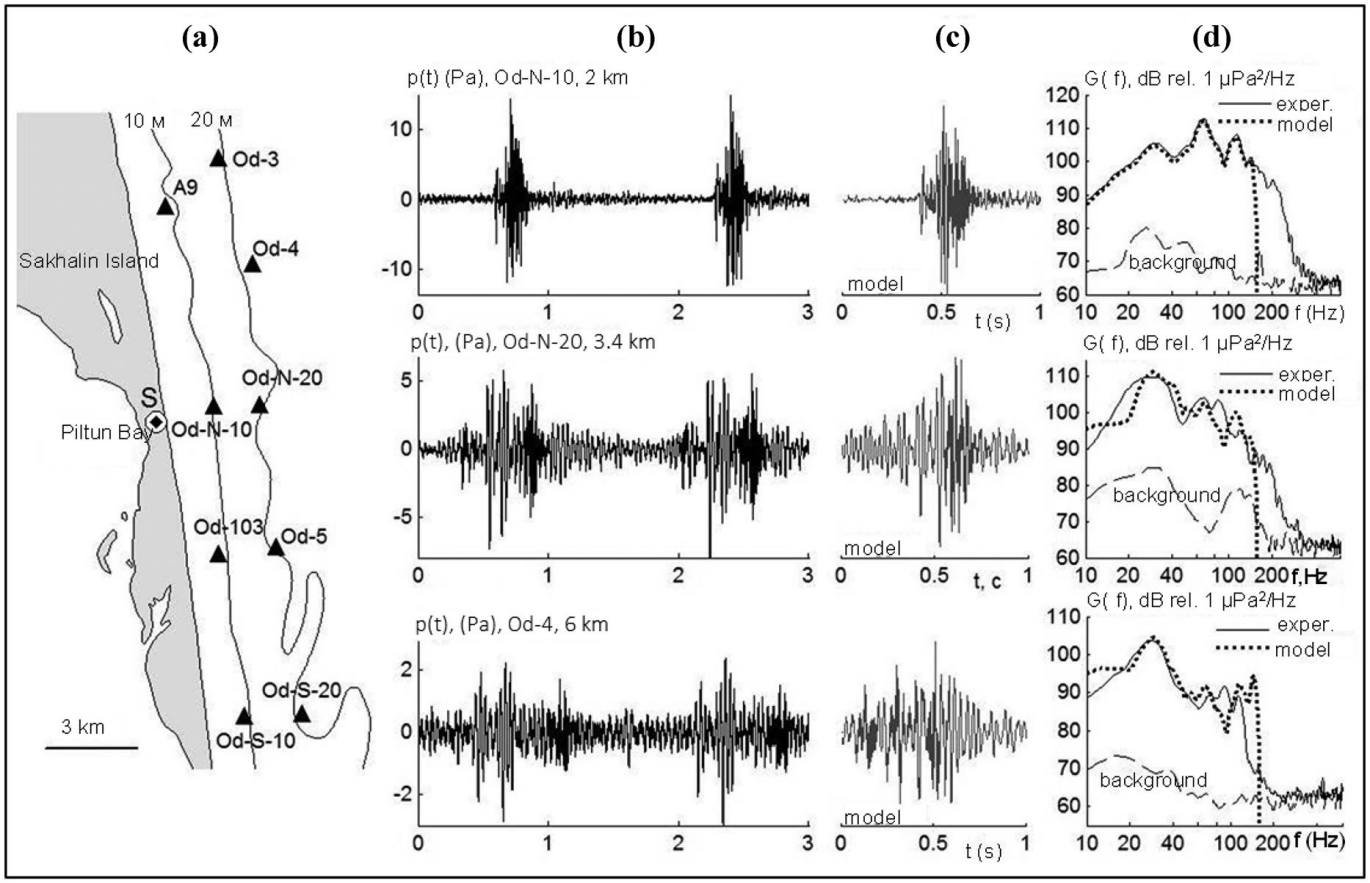
**a** Map of the Odoptu area showing the location of the foundation pile driving (S) and acoustic monitoring locations (triangles) **b** variations in acoustic pressure recorded at the bottom during pile driving **c** corresponding model impulse; and **d** spectra of measured and modeled impulses

Figure 9 shows the location of the onshore site (S) where foundation piles were driven, the acoustic monitoring locations, and the time and frequency domain impulse characteristics.

The elastic parameters of the sub-bottom sediments were selected by comparing experimental and model data at the acoustic monitoring locations. Time-domain plots of impulses (Fig. 9c) were modeled using the MPE approximated with the first three non-interacting vertical modes and a narrow-angle parabolic equation in the horizontal plane incorporating the elastic properties of the seabed rocks (Trofimov et al., 2015).

**Fig. 10:**
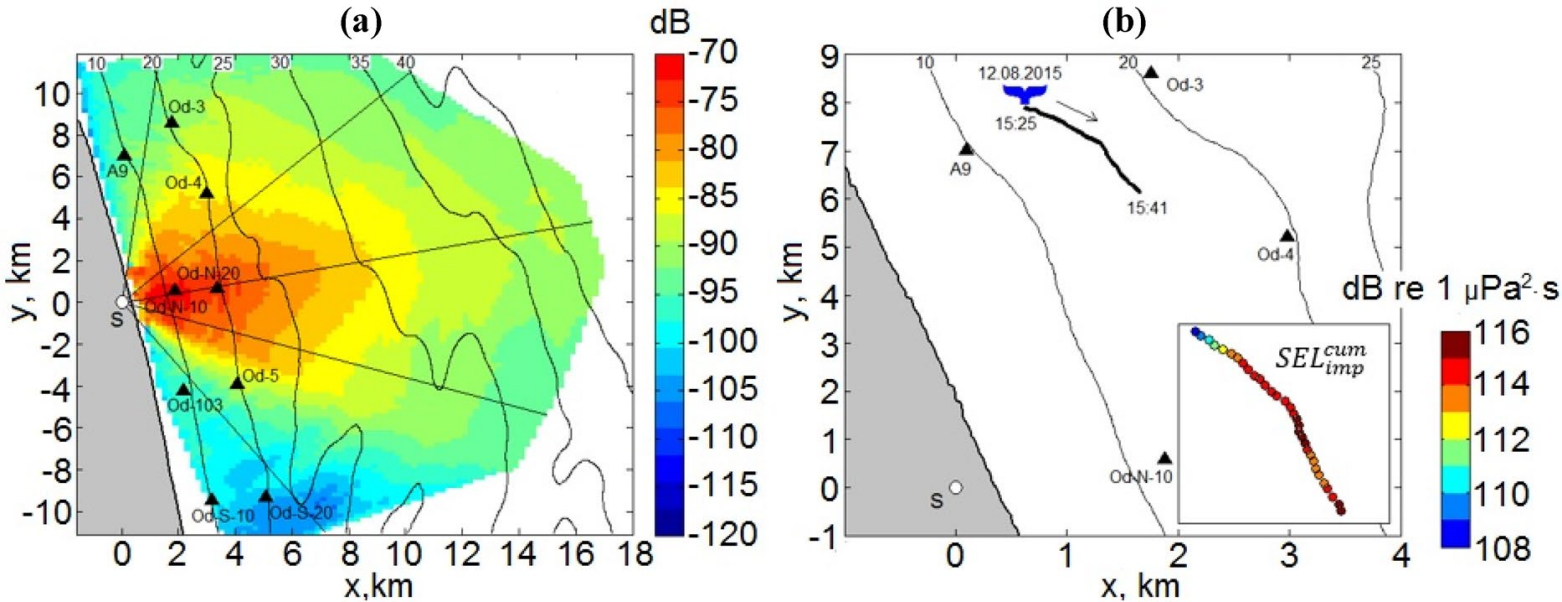
For pile driving, an example of **a** spatial distribution of the median over depth propagation loss for SEL in the water layer and **b** cumulative sound exposure level at 30-s intervals along a behavioral gray whale track

The acoustic field was estimated by interpolating and smoothing the area between the five narrow-angle segments (Fig. 10a). The modeled SEL estimated at the location of the whale was the median-value-over-depth modeled in the frequency range from 10 to 150 Hz. This was computed from the sound exposure source level, which represented the average energy of a blow at the base of the modeled pile. In this case, the sound exposure source level was estimated to be 197.6 dB re 1µPa^2^m^2^s. The level was estimated for each pile at a depth of 20 m using the technique described in Rutenko et al. (2016).

Gailey et al. (2022a) recorded the movement patterns of the whales while they were observed at the surface and interpolated positions of each whale at 30-s intervals as it moved along the shore from north to south (Fig. 10b). The acoustic exposure level due to onshore pile driving at the gray whale location was estimated using two methods.

Acoustic exposure was estimated as the median of the SEL values estimated in the water layer for a specific location at the nodes of a computational grid. For each 30-s location along the whale track, the source function was calculated, and a correction was made to the source level to account for the change in the characteristics of the impulses as the pile is driven deeper into the ground. The SEL values in the water layer for the location of the whale were estimated by adding the median PL between the pile and the whale to the corrected source level for the specific pile strike.

The second method computed the SEL at the whale by estimating the SEL value near the bottom at the nearest AUAR. The impulse characteristics estimated from the automated analysis were averaged for the 30-s intervals tied to the intervals along the whale tracks. A theoretical correction was applied between the closest AUAR and the location of the whale along the track by adjusting for the differential PL between pile location and the two locations. In cases where recorded data were unavailable from the closest AUAR, data from farther AUARs were used.

Values calculated using both methods were compared and found to be similar; both were made available for input to separate analyses of possible acoustic effects on whales. The metric required for these analyses was the cumulative SEL for each 30-s interval; thus, the SEL for a pile strike and the number of pile strikes was provided for each 30-s period.

### Modeling the sound field from vessels

Propeller cavitation and hull vibration caused by internal machinery were the main sources of underwater noise from vessels. Different types of vessels have characteristic source level spectra (i.e., variations of sound emission levels with sound frequency) because of their specific design and operating conditions. For the purpose of modeling noise from a variety of vessels, omnidirectional source level (SL) spectra representative of the mean levels for each vessel class were used (National Research Council (US), 2003). The mean level spectra were then adjusted for vessel speed through water to estimate the effective source level for a vessel in operation. This approach was limited in its ability to account for factors such as hull loading, static pull, thruster use, and other operational conditions that can affect substantially the sound emission of a vessel in activities other than plain transiting; nonetheless, it enables a reasonably realistic modeling of sound exposure from vessels for which only basic specifications may be known and no direct measurement of source levels may exist.

In this study, 125 individual vessels were identified from the marine Automatic Identification System (AIS) and other records as having been present in the region, some for extensive periods and some briefly. Ten SL classes were defined for this analysis. In some cases, the classes were standard; in others, they were derived from direct measurements performed during past operations on vessels regularly active in the region, such as *Britoil 51*, *Katun*, or *Neftegaz 22*, which also could act as proxies for similar tugs and work boats. Each vessel was attributed a specific SL class based on specifications: type, dimensions, and deadweight. Each SL class was assigned specific source level spectrum defined in 1/3-octave bands for reference speed, as well as a scaling factor to account for vessel speed variability.

**Fig. 11:**
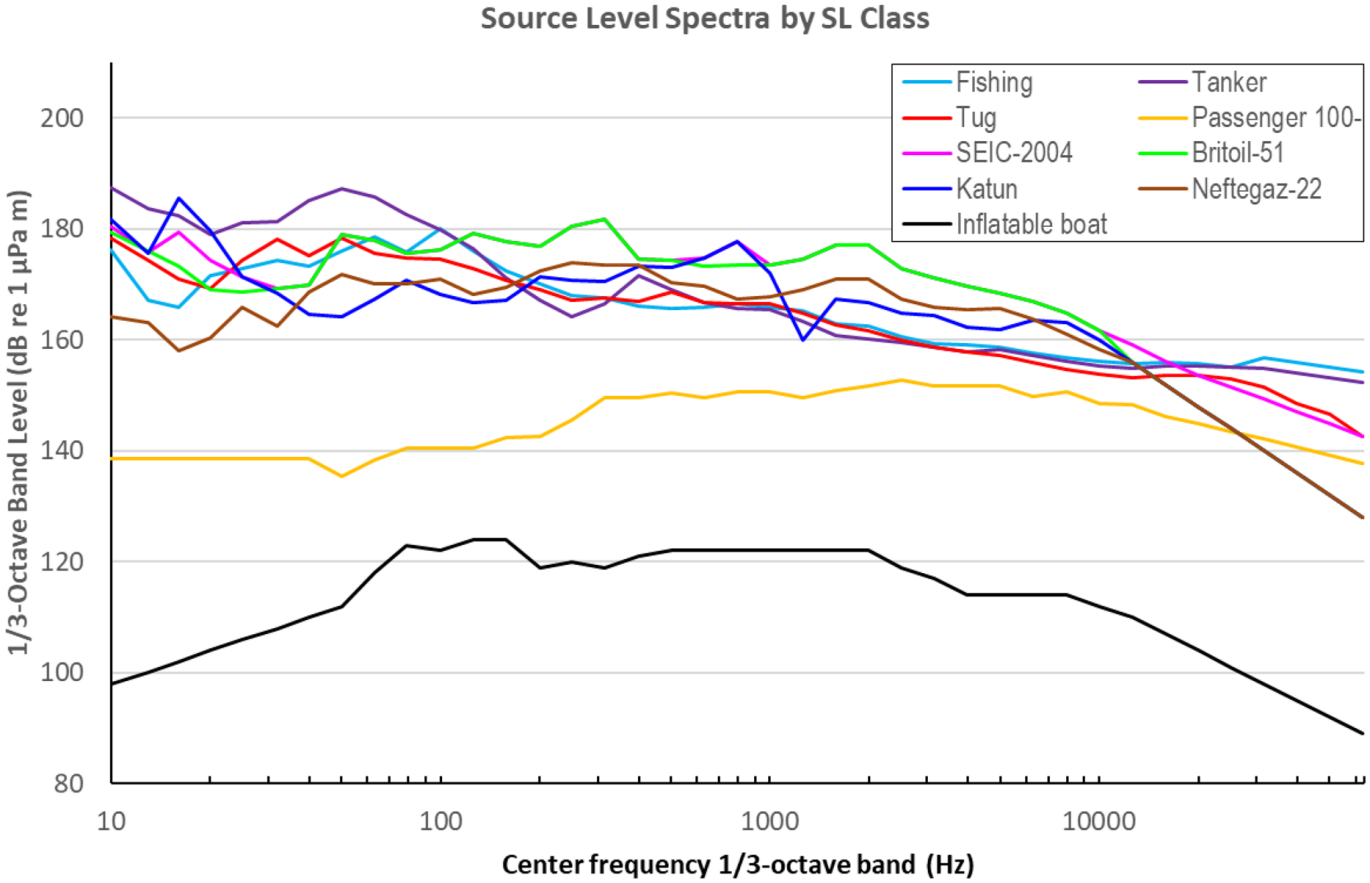
SL spectra by SL class expressed as 1/3-octave band levels versus band center frequency

The base SL spectra for the ten SL classes that were paired with vessel classes in this study are shown in Fig. 11, expressed as 1/3-octave band levels by the center band frequency.

The SL spectrum used in the modeling was the base SL spectrum shown in Fig. 11 for the relevant vessel type adjusted for vessel speed; higher vessel speeds result in higher values for the SL spectrum. If *V* is the vessel speed in m/s and SL_B_(*f*) is the base SL spectrum, the speed-adjusted SL spectrum SL(*f*) is calculated as follows:

1 $$SL(f) = {SL}_{B}(f) + F\;{\mathrm {log}_{10}}(V/{V}_{ref}),$$

where *F* is the “speed factor” and *V*_ref_ is the “reference speed.” This SL scaling with vessel speed is based on a well-established power-law model (Ross, 1976).

As was the case for the modeling of sound from seismic survey sources, a key requirement was an accurate and comprehensive record of the navigational data for all vessels. Here, a validated AIS data set merged from multiple streams provided most of the navigational information; in addition, direct Global Positioning System (GPS) logs downloaded from handheld devices provided time-resolved positional data for the inflatable boats used in various monitoring and support activities throughout the season. Some basic assumptions about vessel positional data were used in bridging gaps and addressing anomalies.

Prior to use in modeling, the navigational data for vessel sound exposure estimation were subjected to a conditioning process that included binning either in time (every 100 s) or in displacement (every 200 m) and averaging within each bin as a means of smoothing out irrelevant jitter and ensuring suitable uniformity of sampling both spatially and temporally. To detect and overcome navigation data deficiencies, vessel velocities were calculated by three methods with different temporal sensitivities: from bin-to-bin positional change, as the average of the AIS-reported velocity for the track points within each bin, and using cumulative point-to-point distance and time difference between first and last point within each bin.

Production modeling of sound exposure from vessels followed essentially the same approach as for seismic sources with one major exception: no measurement-based correction could be applied because reference values for individual vessels could not be derived from sound level measurements at the acoustic stations. Because vessel sound is continuous, only the SEL metric had to be estimated; the SEL accrued over or scaled to a 1-s period would then be numerically equivalent to SPL, with no requirement for the estimation of conversion factors.

For WTE estimation, the 30-s SEL values from the vessels were modeled along individual propagation paths between the vessel positions and the tracked whale locations. The estimation of this cumulative metric was based on the assumption that sound output remained stationary over the 30-s period so that the expression SEL_30sec_ = SPL + 10log(30) could be used. The output generated every 30 s at the whale track point, estimated separately for each vessel that came within 30 km of the whale during that whale tracking period and stored as a data structure, included the following information: vessel coordinates, whale-vessel range, vessel SL, received SPL, and SEL_30sec_.

For GE estimation, the sound exposure was calculated using the same pre-modeled framework described earlier for a seismic source at 5 m depth, which coincided with the notional source depth for all vessel SL classes except for inflatable boats (the latter were easily processed as a special case because of the much more limited range over which their contribution to received levels was of any relevance). The computation was performed in 25-s steps to properly track faster moving vessels; the received level for each computational grid point was obtained from the instantaneous speed adjusted SL and the pre-computed PL framework. The SEL was built up by adding exposure from each 25-s interval and the SPL taken as the maximum from all 25-s intervals. On 5-min boundaries, the sound exposure statistics on the output grid (1 × 1 km cell size) were generated by taking the median and maximum of the SEL and SPL values over the 4 × 4 underlying cells (250 × 250 m size) of the computational grid. The overall period for GE modeling from vessels was from 6 June to 30 September 2015.

### Estimating the ambient noise in the analysis area

The background noise level was estimated from acoustic recordings made at the seafloor with AUARs deployed at the various acoustic monitoring locations, using the SEL calculated over 6-h windows and analyzed in the frequency range from 17 to 14,127 Hz to isolate the effect of flow noise for the entire recording season. To statistically estimate the variation in the 1/3-octave power spectral density, estimates with time and operational activities, power spectral density percentile distribution plots for the minimum; and 10, 25, 50, 75, and 90% and maximum percentiles were generated for the specified analysis period. Once the percentile spectral data had been calculated for all the acoustic locations at which an AUAR was deployed, the 10% spectral values were taken as an analog for the acoustic background.

## Summary

During the summer of 2015, forty autonomous underwater acoustic recorders were used to measure the acoustic field on the northeast Sakhalin shelf and to document the variations in acoustic levels resulting from four seismic surveys and onshore pile driving in the monitoring area. These data recordings were analyzed, and the resulting computed metrics for impulsive noise were used to calibrate acoustic models. These models were, in turn, used to compute specific acoustic variables (1) at 300-s intervals in 1-km^2^ cells of a 4420-km^2^ distribution grid in a gray whale feeding area and (2) at 30-s intervals along known whale tracks. Final results offer, in essence, a dynamic acoustic footprint for a complex industrial operation in an area frequented by feeding gray whales; that is, this work documents relevant acoustic metrics and how they change across time and locations in an operating oil field during seismic operations. Although it is impossible to summarize the full extent of our results here, they will be used in analyses described in Gailey et al. (2022a, b), Schwarz et al. (2022), and elsewhere to assess whale responses to industrial sounds, and, ultimately, to assess the biological relevance of those responses.

## Data Availability

This paper presents a methodological approach rather than the full results of analyzing a dataset; as such there is no pertinent repository of raw or generated data that could be made available for a comprehensive verification. The data underlying specific output presented in this work as examples can be requested from the corresponding author.
